# New tricks for old drugs- praziquantel ameliorates bleomycin-induced pulmonary fibrosis in mice

**DOI:** 10.1186/s40360-024-00737-7

**Published:** 2024-02-14

**Authors:** Yanjun Zeng, Rui Hu, Wei Ma, Ying Ding, Yi Zhou, Xin Peng, Lixin Feng, Qingmei Cheng, Ziqiang Luo

**Affiliations:** 1 Department of Physiology, Xiangya School of Medicine, Central South University, 110 Xiangya Road, Changsha, 410078 China; 2 Department of Geriatric Medicine, Guangzhou First People’s Hospital, School of Medicine, South China University of Technology, Guangzhou, China; 3 Department of Dermatology, The First Affiliated Hospital of Chongqing Medical University, Chongqing, China; 4 Xiangya School of Medicine, Central South University, Changsha, China; 5 Department of Rheumatology and Immunology, Zhengzhou Central Hospital Affiliated to Zhengzhou University, Zhengzhou, China; 6 Hunan Key Laboratory of Joint Degeneration and Injury, Xiangya Hospital, Central South University, 87 Xiangya Road, Changsha, 410008 China

**Keywords:** Praziquantel, Pulmonary fibrosis, Collagen deposition, TGF-β, Macrophage polarization

## Abstract

**Background:**

Pulmonary fibrosis is a chronic progressive disease with complex pathogenesis, short median survival time, and high mortality. There are few effective drugs approved for pulmonary fibrosis treatment. This study aimed to evaluate the effect of praziquantel (PZQ) on bleomycin (BLM)-induced pulmonary fibrosis.

**Methods:**

In this study, we investigated the role and mechanisms of PZQ in pulmonary fibrosis in a murine model induced by BLM. Parameters investigated included survival rate, lung histopathology, pulmonary collagen deposition, mRNA expression of key genes involved in pulmonary fibrosis pathogenesis, the activity of fibroblast, and M2/M1 macrophage ratio.

**Results:**

We found that PZQ improved the survival rate of mice and reduced the body weight loss induced by BLM. Histological examination showed that PZQ significantly inhibited the infiltration of inflammatory cells, collagen deposition, and hydroxyproline content in BLM-induced mice. Besides, PZQ reduced the expression of TGF-β and MMP-12 in vivo and inhibited the proliferation of fibroblast induced by TGF-β in vitro. Furthermore, PZQ affected the balance of M2/M1 macrophages.

**Conclusions:**

Our study demonstrated that PZQ could ameliorate BLM-induced pulmonary fibrosis in mice by affecting the balance of M2/M1 macrophages and suppressing the expression of TGF-β and MMP-12. These findings suggest that PZQ may act as an effective anti-fibrotic agent for preventing the progression of pulmonary fibrosis.

## Introduction

Pulmonary fibrosis is a life-threatening disease characterized by progressive fibrosis, often resulting in end-stage lung disease, respiratory failure, and fatal outcomes. Especially the idiopathic pulmonary fibrosis (IPF), the median survival time is only 3–5 years following diagnosis, and the 5 year survival rate is between 20 and 40% [1]. Pirfenidone and Nintedanib are the two primary approved agents for IPF treatment, mainly for their ability to increase forced vital capacity and slow disease progression in most patients. However, whether these drugs can alleviate symptoms and improve survival rates is still unclear. Moreover, studies indicate that almost all patients treated with Pirfenidone reported at least one adverse event, such as nausea, photosensitivity reactions, and rash [2]. These findings highlight the urgent need to develop safe and effective therapeutic agents for IPF.

Praziquantel (PZQ) is the recognized drug of choice for schistosomiasis, and is generally considered non-toxic with few or transient mild side effects [3]. Previous studies have demonstrated that prolonged PZQ treatment can suppress the development of liver fibrosis in mice with schistosomiasis. Andrade’s group suggested that PZQ’s antifibrotic potential in chronic schistosomiasis patients may depend mainly on its antiparasitic effect [4]. But it was also reported that PZQ inhibited the liver fibrosis caused by non-schistosomiasis factors, such as CCl_4_, by reducing the expression of hydroxyproline, Type I, III, IV collagen, interleukin (IL)-4, IL-13, and transforming growth factor (TGF)-β [5]. These inflammatory factors and cytokines also play significant roles in the development of pulmonary fibrosis.

Therefore, we speculate that PZQ may alleviate pulmonary fibrosis induced by non-schistosomal causes. In this study, we used a bleomycin-induced mouse model of pulmonary fibrosis to investigate the role of praziquantel in non-schistosomal pulmonary fibrosis and explore its potential mechanism, aims to find a new treatment for pulmonary fibrosis.

## Methods

### Animals and drug

Adult male C57BL/6 mice (18–20 g) of specific pathogen-free (SPF) grade were obtained from Hunan SJA Laboratory Animal Co. Ltd. (Hunan, China). The mice were housed in a quiet, antigen‑free environment with free access to food and water, and were maintained on a 12 h light-dark cycle.

PZQ was purchased from Jianglai Biotechnology Co. Ltd. To prepare PZQ injection, we dissolved 100 mg PZQ in 1 ml dimethyl sulphoxide (DMSO), added 7 ml polyethylene glycol (PEG), and mixed the solution thoroughly. The mixture remained stable at room temperature without color or sediment formation changes. Before injection, the solution was diluted with 12 ml normal saline (NS) (DMSO: PEG: NS = 1:7:12, final PZQ concentration: 5 mg/ml).

### Animal experimental protocol

Sixty mice were randomly divided into four groups: (1) control group (CTRL, n = 15); (2) BLM group (BLM, n = 15); (3) BLM + PZQ group (BLM_PZQ, n = 15); (4) normal saline + PZQ group (PZQ, n = 15). Mice in the BLM and BLM_PZQ groups received an intratracheal instillation of bleomycin (5 mg/kg; MedChemExpress, purity 98.81%), while mice in the CTRL and PZQ groups were administered normal saline. Mice in the PZQ and BLM_PZQ groups received PZQ (0.2 ml, bid) by intraperitoneal injection for 21 days after transtracheal injection of bleomycin or normal saline, whereas mice in CTRL and BLM groups were administered solvent. All surgeries were performed under anesthesia using intraperitoneal injection of 1% sodium pentobarbital (0.2 ml/20 g). After 21 days of bleomycin or normal saline administration, mice were sacrificed.

### Histopathological evaluation

Fresh lung tissue was fixed in 4% paraformaldehyde for 24 h and embedded in paraffin. The tissue was cut into 5 μm-thick sections, stained with hematoxylin and eosin (HE) and Masson’s trichrome. The Ashcroft score was used to evaluate the degree of pulmonary fibrosis [6]. Additionally, we used Image J (v1.48, NIH) imaging software to analyze Masson’s three-color digital images. Histopathological examination was performed by an experienced pathologist who was blinded to the groups.

### Hydroxyproline quantification

Hydroxyproline levels in lung tissue homogenates were measured using an assay kit (Beijing Dingguo Changsheng Biotechnology Co. Ltd.) following the manufacturer’s instructions. Sample absorbencies were assessed at 550 nm.

### RT-PCR

RNA was extracted from lung tissue to determine the expression levels of Type I/III collagen and inflammatory mediators, following the manufacturer’s instructions for the kit (RR037A, Takara, Japan). The specific primer sequences used are shown in Table 1. Relative gene expression was analyzed by the 2^−ΔΔCt^ method and was normalized to β-actin mRNA levels.

**Table 1:** Primer sequences

Gene		Sequence
**Procollagen I**	Forward primer	5′-ACAGCAAGGGACTAGCCAGGAG-3′
	Reverse primer	5′-GGAGTGCCTCTTCTGCCAGTTC-3′
**Procollagen** III	Forward primer	5′-GCTCCTCTTAGGGGCCACT-3′
	Reverse primer	5′-CCACGTCTCACCATTGGGG-3′
**TGF-β1**	Forward primer	5′-TCGTGATCGTAGTAGGATAGTCA-3′
	Reverse primer	5′-AGTGTCTAGCTAGCTACATGA-3′
**MMP12**	Forward primer	5′-GAGTAGCACACGCTTTGTTTGT-3′
	Reverse primer	5′-GATAGAAGGCAGACCAGGACAC-3′
**IFN-γ**	Forward primer	5′-TGATCCTTTGGACCCTCTGACTT-3′
	Reverse primer	5′-GCTGGACCTGTGGGTTGTTGA-3′
**IL-4**	Forward primer	5′-GTTGTCATCCTGCTCTTCTTTCTCG-3′
	Reverse primer	5′-CGTGGTACTTACTCAGGTTCAGG-3′
**IL-12**	Forward primer	5′-CCAAGGTCAGCGTTCCAACA-3′
	Reverse primer	5′-AGAGGAGGTAGCGTGATTGACA-3′
**IL-13**	Forward primer	5′-CTCTTGCTTGCCTTGGTGGTCTCG-3′
	Reverse primer	5′-TCCCTCCTCCCAACTCCTCCTTCC-3′
**β-actin**	Forward primer	5′-TGGCCGGGACCTGACAGACTACCT-3′
	Reverse primer	5′-GATGCCACAGGATTCCATACCCAAG-3′

### Cell culture and treatment

Mouse lung fibroblasts were isolated from pulmonary tissue by digestion using a Dulbecco’s modified Eagle’s medium (DMEM, Hyclone, USA)-prepared solution containing collagenase (0.1 U/mL, Worthington, NJ), trypsin (0.125%, Gibco, USA), and DNase I (0.1 U/mL, Thermo Fisher Scientific, USA) [7]. After filtration, cells were resuspended in DMEM with 10% fetal bovine serum (Gibco, USA) and antibiotics (100 U/ml penicillin, 100 U/ml streptomycin), and maintained at 37 °C in a humidified atmosphere of 95% air and 5% CO_2_. One day before the experiment, 5 × 10^3^ cells were seeded into each well of a 96-well plate.

To determine the optimal concentration of TGF-β1 and assess PZQ cytotoxicity, cells were treated with various concentrations of TGF-β1 (0, 0.1, 1, 10, 20, 50 ng/mL; Proteintech, China) and PZQ (0, 0.5, 1, 4, 8, 16, 32 μmol/L). After 48 h of treatment, cell viability was assessed using MTT at OD450. Subsequently, cells were divided into six groups: (1) control group; (2) TGF-β1 group: cells were treated with 10 ng/mL TGF-β1; (3)–(6) TGF-β1 + PZQ groups: cells were treated with different concentrations of PZQ (0.01, 0.1, 1, 10 μmol/L), followed by TGF-β1 (10 ng/mL) 30 min later. After 48 h of TGF-β1 treatment, cell viability was assessed using a CCK8 kit at OD450.

### Bronchoalveolar lavage fluid (BALF)

BALF was collected at the 21 day post-BLM administration. Briefly, mice were anesthetized with intraperitoneal injection of 1% sodium pentobarbital (0.2 ml/20 g). An incision was made in the neck skin to expose the trachea, and a 0.8 mm diameter catheter was inserted into the trachea and fixed, then 0.5 ml precooled phosphate-buffer saline (PBS) was administered into lungs and extracted [8]. This procedure was performed four times. Collected BALF was centrifuged at 1200 × g for 10 min at 4 °C to collect cells. Then cells were resuspended in the wash buffer and used for flow cytometry assay. After sample collection, the mice were sacrificed.

### Flow cytometric method

Red blood cells were lysed with ammonium-chloride-potassium (ACK) solution, and cells were counted using a cell counting chamber. Approximately 1 × 10^6^ cells/ml in 3% PBS solution were stained with a macrophage phenotyping cocktail comprising CD11b-FITC, CD86-PE, F4-80-APC, and CD206-perCP-Cy5.5 (BD Bioscience, USA) for 30 min at 4 °C. Cells were then washed with PBS, resuspended in FACS buffer, and analyzed using a flow cytometer (Beckman Coulter, USA).

### Statistical Analysis

Data were presented as mean ± standard error of the mean (X ± SEM). The significance of the changes was evaluated using a Newman-Keuls Multiple Comparison Test. *P* value < 0.05 was considered statistically significant.

## Results

1. PZQ inhibits lung morphological changes and improves the survival rate of BLM-induced pulmonary fibrosis mice.

**Fig. 1:**
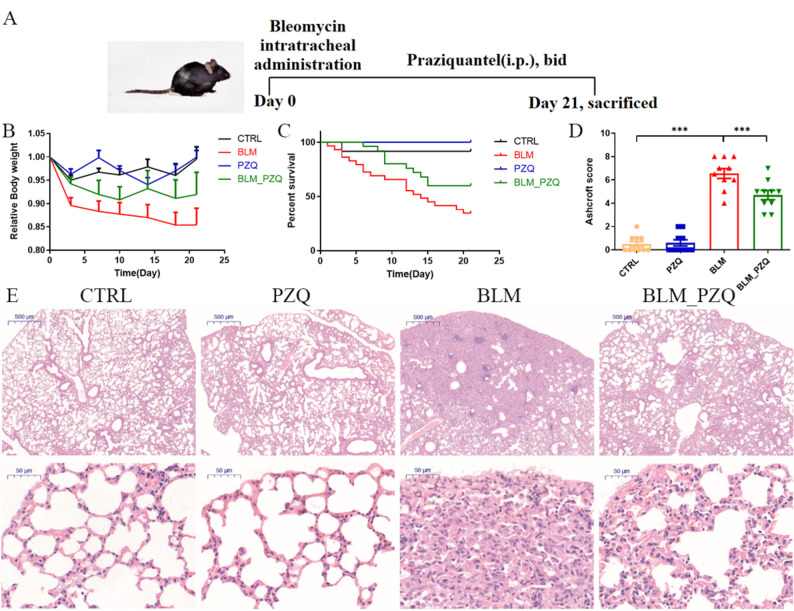
Protective effect of praziquantel on bleomycin-induced pulmonary injury in mice. (**A**) Animal experimental protocol. (**B**) Trends in body weight changes among the different groups of mice. (**C**) PZQ treatment improved the survival rate of mice injected by BLM (*P* = 0.0472) (n = 15). (**D**) Ashcroft scores of histological lung sections. (**E**) Representative histological lung sections stained with HE (bar = 500 μm; bar = 50 μm). All quantification graphs represent mean ± SEM. ***P < 0.001

To investigate the protective effect of PZQ in BLM-induced pulmonary fibrosis, BLM-induced mice were administered with PZQ for 21 days (Fig. 1A). The results showed that the body weight of mice in the BLM group significantly reduced compared with the control group (*P* = 0.0175), treatment with PZQ reduced weight loss caused by BLM (*P* = 0.1891), although the result did not show statistical significance (Fig. 1B). Moreover, PZQ administration improved the survival rate of mice injected with BLM (*P* = 0.0472) (Fig. 1C).

Histological examination using hematoxylin and eosin staining revealed that BLM induced marked structural abnormalities in lung tissue, alveolar septal thickening and infiltration of inflammatory cells. However, treatment with PZQ significantly attenuated lung damageand fibrotic changes induced by BLM (Fig. 1D, E). These findings suggest that PZQ mitigates lung morphological changes and confers a protective effect against BLM-induced pulmonary fibrosis in mice.

2. PZQ reduces collagen deposition in BLM-induced lung fibrosis mice.

**Fig. 2:**
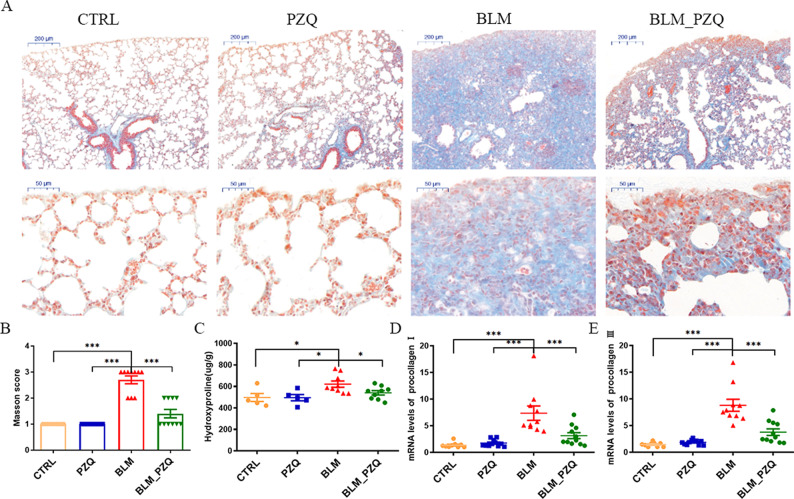
PZQ reduces the collagen deposition of BLM-induced lung fibrosis in mice. (**A**) Representative histological lung sections stained with Masson (bar = 200 μm; bar = 50 μm). (**B**) Masson scores of histological lung sections. (**C**) Hydroxyproline levels of lung tissue. (**D**) Procollagen I mRNA levels of lung tissue. (**E**) Procollagen III mRNA levels of lung tissue. These experiments were repeated three times. All quantification graphs represent mean ± SEM. *P < 0.05. ***P < 0.001

To further clarify the protective effect of praziquantel in the mouse model of pulmonary fibrosis, we evaluated collagen deposition using Masson staining and hydroxyproline levels. As shown in Fig. 2A, B, BLM-induced mice exhibited more extensive collagen deposition in lungs compared to control mice. Moreover, hydroxyproline levels and mRNA expression of collagen I/III were significantly elevated in BLM-induced mice. Treatment with PZQ significantly decreased collagen deposition, hydroxyproline levels, and collagen I/III mRNA expression (*P* < 0.05; Fig. 2C–E). These results suggest that PZQ treatment can reduce collagen deposition in a BLM-induced pulmonary fibrosis mice model.

3. PZQ suppresses transcriptional expression of TGF-β and MMP-12 in BLM-induced pulmonary fibrosis mice.

**Fig. 3:**
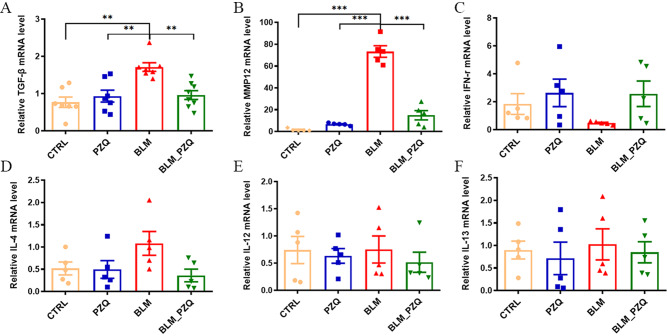
PZQ reduces the levels of TGF-β and MMP-12 in BLM-induced lung fibrosis mice. (**A**) Relative mRNA levels of TGF-β. (**B**) Relative mRNA levels of MMP-12. (**C**) Relative mRNA levels of INF-γ. (**D**) Relative mRNA levels of IL-4. (**E**) Relative mRNA levels of IL-12. (**F**) Relative mRNA levels of IL-13. These experiments were repeated three times. All quantification graphs represent mean ± SEM. **P < 0.01. ***P < 0.001

In our study, we examined some pro-fibrotic cytokines and found that in BLM-induced pulmonary fibrosis mice, the mRNA expression levels of TGF-β and MMP-12 were significantly elevated compared to the control group. However, after treatment with PZQ, these levels were markedly reduced (Fig. 3A, B). While there were no statistically significant differences of the mRNA level of IFN-γ, IL-4, IL-12, and IL-13 in different group mice (Fig. 3C–F). Our data demonstrate that PZQ can effectively suppress the transcriptional expression of TGF-β and MMP-12 in BLM-induced pulmonary fibrosis mice.

4. PZQ inhibits the proliferation of mouse lung fibroblasts induced by TGF-β.

**Fig. 4:**
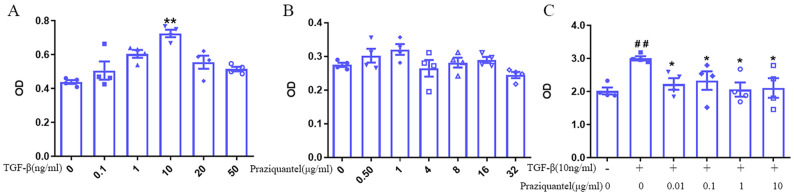
PZQ suppresses the activity of fibroblast in vitro. (**A**) OD of mouse lung fibroblasts treated with TGF-β (0, 0.1, 1, 10, 20, 50 ng/ml) measuring by MTT. (**B**) OD of mouse lung fibroblasts treated with PZQ (0, 0.05, 1, 4, 8, 16, 32 μg/ml) measuring by MTT. (**C**) OD of mouse lung fibroblasts treated with TGF-β (0, 10 ng/ml) and PZQ (0, 0.01, 0.1, 1, 10 μg/ml) for 48 h measuring by CCK-8. #compared with cells treated without TGF-β or PZQ; *compared with cells only treated with TGF-β. These experiments were repeated three times. All quantification graphs represent mean ± SEM. ##*P* < 0.01. *P < 0.05. **P < 0.01

To investigate the direct effect of PZQ on fibroblast proliferation, first, we conducted an MTT assay on mouse lung fibroblasts treated with different concentrations of TGF-β1 for 48 h to determine the appropriate concentration of TGF-β1 to activate fibroblasts. We found that treatment with 10 ng/mL TGF-β1 significantly increased fibroblast proliferation (Fig. 4A). Next, we treated mouse lung fibroblasts with various concentrations of PZQ (0, 0.05, 1, 4, 8, 16, 32 μg/mL) for 48 h and found no cytotoxicity with PZQ treatment (Fig. 4B). Finally, we treated mouse lung fibroblasts with 10 ng/mL TGF-β1 and varying concentrations of PZQ (0.01, 0.1, 1, 10 μg/mL) for 48 h and determined the proliferation using a CCK-8 assay. Interestingly, we found that PZQ treatment significantly suppressed the proliferation of fibroblasts induced by TGF-β (Fig. 4C). These results suggest that PZQ might have anti-fibrotic effects by inhibiting the proliferation of lung fibroblasts.

5. PZQ may affect the polarization of macrophages.

**Fig. 5:**
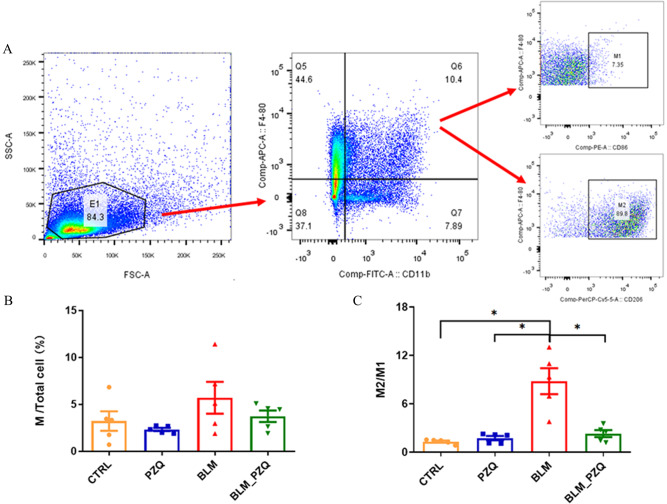
PZQ may protect against BLM-induced lung fibrosis by affecting the polarization of macrophages. (**A**) The rate of M1 and M2 was evaluated by flow cytometry. (**B**) The rate of M/total cells in BALF. (**C**) The rate of M2/M1 in BALF. M: macrophage; M1: type 1 macrophage; M2: type 2 macrophage. These experiments were repeated three times. All quantification graphs represent mean ± SEM. *P < 0.05

Given that M2 macrophages are the major source of TGF-β1 and PZQ can reduce the level of TGF-β in BLM-induced pulmonary fibrosis mice, we hypothesized that PZQ may protect against BLM-induced pulmonary fibrosis by affecting macrophage polarization. To test this hypothesis, we collected macrophages from BALF of mice and analyzed their phenotype using flow cytometry (Fig. 5A). As shown in Fig. 5B, the ratio of M/total cells showed a trend toward an increase in BLM-treated mice compared to controls, which was reduced by PZQ treatment, although this difference was not statistically significant. Moreover, the M2/M1 ratio was significantly increased in BALF of BLM-treated mice, but PZQ treatment decreased the M2/M1 ratio (Fig. 5C). These results suggest that PZQ may affect macrophage polarization, potentially contributing to its anti-fibrotic effects in BLM-induced pulmonary fibrosis.

## Discussion

In summary, this study has demonstrated several key findings: (1) PZQ has a protective effect on BLM-induced pulmonary fibrosis mice. (2) PZQ can ameliorate BLM-induced pulmonary fibrosis in mice by inhibiting the expression of TGF-β and MMP-12. (3) PZQ can directly inhibit the proliferation of fibroblasts induced by TGF-β in vitro. (4) PZQ can reduce the balance of M2/M1 in BALF of BLM-induced mice. These results suggest that PZQ could be a potential new therapeutic approach for treating pulmonary fibrosis diseases.

Pulmonary fibrosis is a life-threatening disease with an aggressive course, depressed life quality, and high mortality [1]. The current understanding of the pathogenesis of pulmonary fibrosis involves recurrent micro-injuries to alveolar epithelial cells in combination with accelerated aging of these cells in genetically susceptible individuals. This leads to aberrant wound healing characterized by the recruitment, proliferation, and differentiation of fibroblasts into myofibroblasts, which subsequently deposit excessive amounts of extracellular matrix (ECM). This process is promoted by pro-fibrotic mediators such as TGF-β [5].

Liang Y. et al. reported that PZQ-prolonged administration had effects of anti-liver fibrosis on mice infected with *S. japonicum* [5]. In addition, it has been found that praziquantel also inhibits the hepatic fibrosis induced by carbon tetrachloride, suggesting that praziquantel may have a general improvement in liver fibrosis caused by multiple injuries [9]. Studies have shown that PZQ is also effective for *S. japonicum* in the skin and lungs, especially in the pulmonary infection stage [10]. The mechanism may be that PZQ can suppress the formation of soluble egg antigens and inhibit the release of inflammatory cytokines in *S. japonicum* eggs, which inhibits the formation of egg granuloma by suppressing the hyperplasia of inflammatory cells such as neutrophils, eosinophils, macrophages and fibroblasts [10]. Moreover, PZQ is reported to prevent the development of pulmonary arterial hypertension associated with *S. mansoni* infection [11] and reverse the changes of severe pulmonary vascular remodeling associated with perivascular inflammation by reducing mRNA levels of inflammatory cytokines IL-13, IL-8, and IL-4 in lungs [12]. These inflammatory cells and cytokines also play an important role in the development of pulmonary fibrosis [13]. However, the current researches all focus on that PZQ can suppress the formation of *S. japonicum* egg granuloma and fibrosis, whether PZQ can inhibit the pulmonary fibrosis caused by non-schistosomiasis factors remains unknown. The current study showed that PZQ can inhibit inflammatory infiltration and collagen deposition in BLM-induced pulmonary fibrosis mice, suggesting that PZQ may have therapeutic potential for treating non-schistosomiasis-related pulmonary fibrosis.

The TGF-β signaling pathway is known to play a critical role in cellular processes such as proliferation, apoptosis, and fibrogenesis [14]. Previous studies have shown that TGF-β gene expression decreased in praziquantel-cured mice compared with untreated controls, which led to reduced expression of hydroxyproline and Type I/III collagen [15, 16]. Similarly, in our current study, we found that PZQ can protect against bleomycin-induced pulmonary fibrosis by suppressing TGF-β expression. Additionally, TGF-β can promote the proliferation of fibroblasts, which is a key driver of fibrogenesis in the lungs. Our results also suggest that PZQ can inhibit the proliferation of fibroblasts induced by TGF-β in vitro, further supporting its potential anti-fibrotic effect.

Matrix metalloproteinase (MMPs) can cut large collagen fibers into segments, promote scar formation and fibrosis regression [17]. Some studies revealed that the expression of MMPs increased in the plasma of IPF patients, which enhanced matrix degradation via the up-regulation of inflammatory mediators. Additionally, some studies have shown that loss of MMP-12 can protect mice from smoke-induced lung disease [18] and FAS-induced pulmonary fibrosis [19]. In our current study, we found that the level of MMP-12 significantly increased in BLM-induced pulmonary fibrosis mice, while treatment with PZQ was able to decrease the level of MMP-12. These findings suggest that PZQ may have anti-fibrotic effects by inhibiting the expression of MMP-12.

Macrophages play a key role in the development of pulmonary fibrosis by secreting proinflammatory cytokines and profibrotic mediators that induce the proliferation and activation of myofibroblasts [20]. During environmental changes, macrophages can polarize into M1 or M2 forms [21]. M1 macrophages promote the initial inflammatory response during the early stage of pulmonary fibosis, whereas M2 macrophages mediate tissue remodeling [22]. Evidence indicates that the M2 phenotype, as opposed to the M1 phenotype, prevails in the lung during the progression of IPF [23]. M2 macrophages are known to be the primary source of key profibrotic mediators such as TGF-β [24, 25]. In another study, the amount of M1 macrophage was upregulated and fibrosis was reduced in the M2 macrophage-suppressed animal model. This means that the balance of the two types of macrophages affects the outcome of the response to fibrotic stimulation [26]. Our data showed a remarkable increase in the M2/M1 ratio in BALF of BLM-treated mice, and PZQ treatment can decrease the M2/M1 ratio. These results suggest that PZQ may affect macrophage polarization, thus alleviating BLM-induced pulmonary fibrosis.

IL-4 and IL-13 are crucial factors in M2 macrophage activation [22]. Our study found a trend of increased IL-4 and IL-13 expression in BLM-treated mice, while treatment with PZQ resulted in reduced mRNA expression levels of IL-4 and IL-13, although this difference was not statistically significant. Further investigation is needed to determine how PZQ affects the polarization of macrophages and the potential mechanisms underlying its anti-fibrotic effects in pulmonary fibrosis.

## Conclusion

In conclusion, this study shows that praziquantel can ameliorate BLM-induced pulmonary fibrosis in mice by affecting the balance of M2/M1 macrophages and suppressing the expression of TGF-β and MMP-12. These results suggest that PZQ may have potential as a new therapeutic approach for treating pulmonary fibrosis diseases. However, further investigation is required to better understand the molecular mechanisms underlying these effects.

## Data Availability

The datasets used and/or analyzed during the current study are available from the corresponding author on reasonable request.
